# Autoimmunity and Gastric Cancer

**DOI:** 10.3390/ijms19020377

**Published:** 2018-01-26

**Authors:** Nicola Bizzaro, Antonio Antico, Danilo Villalta

**Affiliations:** 1 Laboratorio di Patologia Clinica, Azienda Sanitaria Universitaria Integrata, 33100 Udine, Italy; nic.bizzaro@gmail.com; 2 Laboratorio Analisi ULSS 4, 36014 Santorso, Italy; antonio.antico@aulss7.veneto.it; 3 Immunologia e Allergologia, Presidio Ospedaliero S. Maria degli Angeli, 33170 Pordenone, Italy

**Keywords:** autoimmune diseases, autoimmune gastritis, gastric cancer, *Helicobacter pylori* infection, intrinsic factor antibodies, parietal cell antibodies

## Abstract

Alterations in the immune response of patients with autoimmune diseases may predispose to malignancies, and a link between chronic autoimmune gastritis and gastric cancer has been reported in many studies. Intestinal metaplasia with dysplasia of the gastric corpus-fundus mucosa and hyperplasia of chromaffin cells, which are typical features of late-stage autoimmune gastritis, are considered precursor lesions. Autoimmune gastritis has been associated with the development of two types of gastric neoplasms: intestinal type and type I gastric carcinoid. Here, we review the association of autoimmune gastritis with gastric cancer and other autoimmune features present in gastric neoplasms.

## 1. Introduction

Immune dysregulation is believed to play a pathogenic role in the development of both autoimmunity and neoplasia, and autoimmune conditions have been described in patients with neoplastic diseases. Antinuclear antibodies, the hallmark of many autoimmune rheumatic diseases, have been reported in the sera of patients with malignant tumors [1,2,3]; anti-La antibodies which are characteristically detected in sera of patients with Sjӧgren’s syndrome, and anti-CENP-B antibodies, a marker of systemic sclerosis, were detected in patients with breast cancer [4,5]. Similarly, anti-dsDNA antibodies which are of both diagnostic and prognostic value in systemic lupus erythematosus (SLE), were also reported to be present in the sera of patients with various types of cancer [6,7]; the presence of rheumatoid factor was found to correlate with poor prognosis in different types of neoplastic diseases including gastrointestinal cancer [8]. Also, organ-specific antibodies were reported in malignancies; among these are anti-smooth muscle antibodies, anti-parietal cell antibodies and anti-thyroid antibodies [9,10].

Conversely, an increased incidence of malignancies has been observed among patients with autoimmune diseases [11]. According to the Bradford Hill postulates [12] that evaluate the degree in which an autoimmune disease is conditioning a higher probability to develop a malignant neoplasm, a link has been found for rheumatoid arthritis, SLE, Sjӧgren’s syndrome and celiac disease in association with lymphoproliferative diseases [13,14]; idiopathic inflammatory myositis with solid tumors [15]; and systemic sclerosis in association with breast and gastrointestinal cancer [16]. In addition, recent research has shown that neoplastic transformation of autoimmune gastritis is as high as 10% and that autoimmune gastritis should be considered a pre-neoplastic disorder with an annual incidence of gastric cancer of 0.3% [17].

Here, we review the association of autoimmune gastritis with gastric cancer and other autoimmune features present in gastric neoplasms.

### 1.1. Autoimmune Gastritis

Autoimmune gastritis (AIG) is an organ-specific disease characterized by a chronic inflammation of the mucosa of the stomach that evolves in atrophic gastritis causing malabsorption of essential elements and eventually microcytic iron-deficient anemia [18] or pernicious anemia due to vitamin B_12_ deficiency [19]. As the lesion progresses, the parietal and principal cells of the mucosa may be replaced by cells containing mucus, similar to the intestinal ones. Two types of metaplasia are considered to be associated with gastric carcinogenesis in humans: intestinal metaplasia, and spasmolytic polypeptide-expressing metaplasia (SPEM). Goblet cells in intestinal metaplasia express appropriate intestinal markers, including Muc2 and Trefoil factor 3 (TFF3), while the mucous metaplastic lineages in SPEM display morphological characteristics more typical of deep antral gland cells or Brunner’s glands, with expression of Muc6 and Trefoil factor 2 (TFF2). Importantly, recent investigations support the origin of SPEM through transdifferentiation from mature principal cells following parietal cell loss [20]. Both intestinal metaplasia and SPEM have been associated with the progression to intestinal-type gastric cancer [21].

Similar to other autoimmune conditions, AIG is more common in females than in males (3:1 ratio). AIG is generally asymptomatic up to an advanced stage of atrophy and/or dysplasia of the mucosa [22]. For this reason, AIG is a frequently underdiagnosed disease, with an estimated prevalence of nearly 2% in the third decade to 12% in the eighth decade [17,23,24]. The prevalence is even higher in patients affected by other autoimmune diseases, especially autoimmune thyroid diseases (AITD) and type 1 diabetes (T1DM) [25,26]. These associations define the multiple autoimmune diseases (MAS) type 3B and 4 [27].

Chronic autoimmune gastritis (type A) is etiologically and histologically distinct from type B gastritis associated with *Helicobacter pylori* (*H. pylori*) infection [28]. Different from *H. pylori* gastritis which is mainly localized in the antrum, AIG is restricted to the gastric body and fundus because inflammatory aggression affects the cells of the oxytocin glands [29]. However, there is a peculiar form of AIG that may develop in genetically predisposed subjects during *H. pylori* infection [30]. The finding of anti-parietal cell antibodies in 20–30% of patients with *H. pylori* infection and of anti-*H. pylori* antibodies in patients with AIG, suggests that there is a link between *H. pylori* and gastric autoimmunity [31,32,33]. 

*H. pylori* infection could induce AIG through mechanisms of molecular mimicry and/or epitope spreading; a high homology has been demonstrated between the β subunit of Hp urease and the subunit β of gastric ATPase [34]. The activation of gastric Th1 cells reactive to different peptides of *H. pylori* wall that cross-react with gastric H^+^K^+^-ATPase, results in an inflammatory process in which T-cell-derived IFN-γ enables parietal cells to act as APCs and to become targets of cross-reactive epitope recognition resulting in killing or apoptotic suicide. Apoptotic parietal cells would thus allow cross-priming of T cells that are specific to private gastric ATPase epitopes [35,36].

Although histological healing of the mucosa of the body has been reported in patients in whom *H. pylori* had been eradicated [37,38], a direct correlation between *H. pylori* infection and AIG remains controversial [39,40,41]. To this end, it has to be noted that while the bacterium is present in the initial stages of gastritis, in the atrophic stage the bacterium is no longer recognizable because hypocloridry and mucosal destruction result in environmental conditions unsuitable for *H. pylori* survival.

### 1.2. Cell-Mediated Autoimmunity

In AIG, cell-mediated autoimmunity plays a primary role sustained by CD4^+^CD25^−^ Th1 resting lymphocyte effectors [42]. Most of these self-reactive cells produce IFN-γ and TNF-α and possess cytolytic capacities, with perforin and Fas/Fas ligand-mediated mechanisms, which they express in well-defined gene restriction conditions dictated by the MHC system [43]. They induce gastric parietal cell death by apoptosis and perforin/granzyme B pathway, in particular through IFN-γ, which increases the expression of Fas and MHC class II molecules on gastric parietal cells. 

The evidence that in the guinea pigs a single injection of an IFN-γ neutralizing antibody prevents the development of gastritis makes it clear that this cytokine is active in the genesis of the disease [44].

Moreover, the role of CD4^+^CD25^−^ Th1 lymphocytes in the pathogenesis of AIG has been demonstrated by their isolation in the paragastric lymph nodes in experimental murine models and the development of atrophic gastritis with appearance of parietal cell antibodies in association with a decrease in CD4^+^CD25^+^ T-cell tolerance [45].

The main target of immunological injury is the gastric H^+^/K^+^-adenosine-triphosphate enzyme (ATPase), a protein of the membrane that coats the secretory canaliculi of the parietal cells and is responsible for the secretion of hydrogen ions in exchange for potassium ions (proton pump) [46,47]. The gastric H^+^/K^+^-ATPase is formed by a catalytic 100 kDa α subunit and a 60–90 kDa β subunit; CD4^+^ T cells react to H^+^/K^+^-ATPase α chain and marginally to the β chain. Induced by a triggering factor not yet entirely identified, the CD4^+^CD25^−^ T-cells, together with macrophages and B lymphocytes, infiltrate the submucosa, the lamina propria and the gastric glands causing the loss of parietal, principal and P/D1 ghrelin-producing cells [48,49], the principal and P/D1 cells being destroyed as bystanders of the parietal cells.

### 1.3. Humoral Autoimmunity

Patients with AIG have been shown to have two types of antibodies, one to parietal cells (PCAs) and the other to intrinsic factor (IFA) or its binding site in the small bowel.

PCAs are present at a high frequency in AIG (80–90%), especially in early stages of the disease [50,51] and bind to both α and β subunits of gastric H^+^/K^+^-ATPase. Antibody reactivity to the α catalytic subunit includes epitopes on the cytosolic side of the secretory membrane. Antibody reactivity to the β subunit requires that the antigen is linked in a disulfide-bond and glycosylated, thus, suggesting that autoepitopes are located in the luminal domain of the glycoprotein [47,52].

In the later stages of the disease, the incidence of PCA decreases due to the progression of atrophy and the loss of gastric parietal cells and, thus, the decrease in antigenic rate [53,54]. It is currently unknown if these autoantibodies play a pathogenic role in AIG but their finding in serum in the subclinical stage, especially in patients with autoimmune endocrine disease, is predictive of the presence of AIG [55].

Human intrinsic factor (IF) is a 60-kDa glycoprotein secreted by gastric parietal cells. Its action is high affinity binding and transport of vitamin B_12_. The complex IF-vitamin B_12_ reaches terminal ileum where it is absorbed after binding to specific receptors in the membranes of cells of ileal lumen [56]. IFAs are considered specific markers for AIG and are present both in blood serum and in the gastric juice of 30–50% of AIG patients [57]. In serum, two specific types of IFA, both of the IgG class, have been described: type 1 (blocking antibodies) that react with the binding site for vitamin B_12_ and are found in 70% of IFA-positive patients, and type 2 (binding or precipitating antibody) that recognizes a site away from B_12_ binding sites and impedes binding of IF-vitamin B_12_ to the receptors in the ileal mucosa. Type 2 IFAs are found in about 30% of AIG patients, and are rarely present in the absence of type I autoantibodies [58].

## 2. Autoimmune Gastritis and Gastric Cancer

The incidence of gastric neoplasms is higher in patients with AIG compared to the general population [59,60]. Prospective studies have shown that 4–9% of patients with AIG, or its more severe form pernicious anemia, have gastric carcinoid tumors, whose frequency is 13-times higher than that of control subjects [44]. In addition, AIG progression to atrophic gastritis, associated with intestinal metaplasia, may predispose to gastric adenocarcinoma in more than 10% of patients [44]. 

Two recent studies, one with over 4.5 million adult male veterans admitted to US Veterans Affairs hospitals in the United States [61] and the other including nine million individuals from Sweden [62], reported that individuals with AIG/pernicious anemia had a three-fold increased risk of developing not only stomach carcinoid and adenocarcinomas, but also small intestinal adenocarcinomas and esophageal squamous cell carcinomas.

Nguyen and coworkers [63], using a transgenic mouse model of AIG, investigated the potential link between AIG and gastric cancer using CD4^+^ T cells expressing a T-cell receptor specific for a peptide from the gastric H^+^/K^+^ ATPase proton pump. By 2–4 months of age, all mice developed chronic gastritis that resulted from large numbers of CD4^+^ T cells that infiltrated the gastric mucosa and produced large amounts of IFNγ and smaller amounts of IL-17. At this stage of the disease, mice also developed several molecular features similar to those that precede gastric cancer in humans, including SPEM.

**Figure 1:**
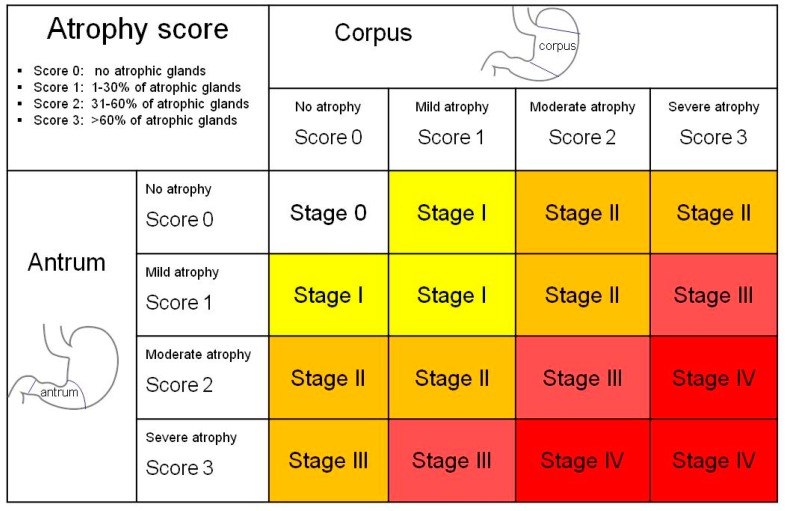
OLGA (operative link for gastritis assessment) staging system for gastritis. Modified from Rugge M. et al. [65].

For these reasons, autoimmune gastritis should be considered a precancerous lesion, and the European MAPS (Management of Precancerous Conditions and Lesions in the Stomach) guidelines [64] recommend a three-yearly endoscopic and bioptic follow-up for all patients with extensive atrophy (stage III and IV of the OLGA classification [65]) (Table 1 and Figure 1).

Gastric atrophy is a key step towards gastric neoplasms, as studies of resected stomachs from patients with intestinal-type gastric cancer have shown gastric atrophy in every case [66]. Atrophy and metaplasia (including SPEM), occur in a setting of inflammation and a complex milieu of cytokines [67]. Studies in humans and mouse models of gastritis and gastric cancer identified important roles for cytokines in regulating oxyntic atrophy, hyperplasia, metaplasia, and progression to gastric cancer. Several reports showed that IL-17A promotes tumorigenesis. In particular: (a) level of IL-17 mRNA in gastric tumors was associated with the depth of tumor, lymph-vascular invasion and lymph node involvement [68]; (b) gastric cancer patients have higher levels of IL-17 in serum and in cancer tissues than the general population [69]; (c) genetic data show that IL-17A and IL-17F polymorphisms increase gastric cancer risk [70]; (d) there are increased Th17 cells infiltrating tumors on patients with advanced gastric cancer [71].

Kuai and coworkers have demonstrated that tumor cells produce IL-8, a cytokine of the CXC chemokine family, as an autocrine growth factor, which promotes tumor growth, tissue invasion, metastatic spread and chemoresistance of gastric cancer cells [72]. Genotypes of TNF, IL10, IL1B, and the interleukin-1 receptor antagonist (IL-1RA) are also reported to confer greater risk of gastric cancer [73]. IL-1β was able to directly induce DNA methylation, which may link inflammation-induced epigenetic changes and the development of gastric diseases [74]. Several additional cytokines (IL-22, IL-23, IL-32, IL-33) have been also implicated in gastric cancer progression [75,76,77]. Taken together, these findings show that diverse cytokines and different combinations of cytokines might promote gastric oncogenesis and/or metastasis. The risk may depend on the types of cytokines made by different subsets of differentiated CD4^+^ helper T cells responding to *H. pylori* or self-antigens such as H^+^/K^+^ adenosine triphosphatase (ATPase) in the case of autoimmune gastritis [73].

However, more information on cytokines that influence gastric cancer development is needed, in particular in light of the development of new biological entities for targeting specific cytokines. In fact, a better understanding of the cytokine pathway promoting gastric cancer development and progression may be used to obtain additional therapeutic options for patients with chronic atrophic gastritis and gastric cancer.

Overall, AIG has been associated with the development of two types of gastric neoplasms: intestinal type and type I gastric carcinoid [78].

### 2.1. Intestinal-Type Gastric Cancer

As previously mentioned, the two known factors predisposing gastric cancer in patients with AIG are intestinal metaplasia and concurrent *H. pylori* infection, which is the most common cause of intestinal metaplasia of the gastric mucosa [79]. It should be noted that *H. pylori* eradication in patients with precancerous lesions (gastric atrophy, intestinal metaplasia or gastric dysplasia) does not significantly reduce the incidence of gastric cancer [80]. However, not all patients with *H. pylori* gastritis develop gastric cancer. Chances are higher when there are some virulence factors. For example, *H. pylori* cagA-positive strains have been shown to pose a significantly greater risk of developing peptic ulcers and gastric cancer than cagA-negative strains [81,82]. Another well-known virulence factor is the vacuolating cytotoxin A (vacA) protein [83].

The pathway of gastric cancer development, mainly of the intestinal histological type, was described by Correa [84]: chronic inflammation leads to tissue atrophy, which is further followed by intestinal metaplasia. Unknown genetic, metabolic or environmental triggers eventually lead to the development of adenocarcinoma. In a recent systematic review, an annual incidence of gastric adenocarcinoma of 0.27% per person-year was demonstrated, with an overall relative risk of 6.8 [60]. In another study, in which 877 Danish patients with gastric cancer were examined, 12 (1.3%) had a previous diagnosis of AIG [85]. According to the typical distribution of lesions in AIG, these tumors were localized to the body and to the fundus of the stomach, while they were mainly affecting the antral and pyloric region in patients without AIG (*H. pylori* infection was not investigated).

### 2.2. Type I Gastric Carcinoid

Hypergastrinemia resulting from the loss of HCl secretion by gastric parietal cells leads to the development of hyperplasia of the enterochromaffin cells with possible evolution into a carcinoid tumor. Carcinoid tumor in patients with AIG represents about 10% of all carcinoid tumors and about 1% of gastric neoplasms [86,87].

There are three types of gastric carcinoid characterized by different levels of gastrin: (a) type I associated with a very high gastrinemia resulting from AIG; (b) type II which is present in patients with multiple endocrine neoplasia (MEN) and show elevated levels of gastrin; (c) type III presenting as Zollinger–Ellison syndrome which is the most aggressive variant and showing a normal gastrin level [88]. In type I carcinoid, lesions are characterized by the secretion of gastrin in response to the loss of the negative feedback due to the loss of parietal cells, which produce hydrochloric acid. Hypergastrinemia, in turn, has trophic effects on enterochromaffin cells. Hyperplasia and subsequent dysplasia of enterochromaffin cells may progress toward the gastric carcinoid type I over time [89]. In addition, chronic achlorhydria increases the production of gastrin by the G cells in the antrum, which then stimulates enterochromaffin cells that lead to their hyperplasia. Patients with type I gastric carcinoid are generally asymptomatic, although dyspeptic symptoms may be present. For this reason, diagnosis is usually performed during endoscopic examination [90]. 

### 2.3. Cancer Stem Cells

Recently, a cancer stem/initiating cell concept was proposed to explain cancer development. 

According to Visvader [91], either stem or progenitor cells can act as targets for tumor initiation. Several diverse cancers are hierarchically organized and sustained by a subpopulation of self-renewing cells that can generate the full repertoire of tumor cells (both tumorigenic and non-tumorigenic cells). Stem cells have been favored candidates for targets of transformation because of their inherent capacity for self-renewal and their longevity, which would allow the sequential accumulation of genetic or epigenetic mutations required for oncogenesis.

Indeed, it has been demonstrated that, as one of the possible mechanisms of gastric carcinogenesis, chronic inflammation induced by *Helicobacter pylori* infection can increase the number of tissue stem/progenitor cells, promote their proliferation, and alter the properties of stem cells toward intestinal metaplasia to cancer [92]. Thus, an intestinal phenotype in the stomach would be not just a differentiated metaplasia in the stomach, but a phenotype of stem cell abnormality with precancerous lesion susceptible to gastric carcinogenesis after chronic inflammation [92].

## 3. Autoantibodies as Markers of Gastric Cancer

Cancer cells can induce an immunological response resulting in the production of autoantibodies against tumor antigens which can be used as biomarkers to detect cancer at an early stage. Indeed, the immune system is capable of sensing at least some tumor-associated antigens before many standard clinical tests for cancer diagnosis [93], so that detection of tumor-associated autoantibodies could have both diagnostic and prognostic relevance [94,95]. Availability of early and specific markers would be an important advance in cancer management because currently a significant proportion of individuals are diagnosed late, presenting with advanced disease at which time the opportunities for successful treatment are drastically reduced and treatment costs significantly increased [95]. The use of autoantibodies as biomarkers in cancer immunodiagnosis is further justified by the fact that these antibodies are generally absent or present in very low concentration in normal individuals and in non-cancer conditions [96]. Importantly, although no evidence of correlation between antibody concentration and cancer stage emerged from most studies [94], usually a marked decrease in antibody levels is seen after surgical removal of solid tumors, indicating that they can be used in monitoring the efficacy of surgical treatment and in patient follow up.

Most tumor-associated autoantigens are cellular proteins and belong to three main classes: (a) antigens resulting from genetic mutations or rearrangements; (b) viral antigens; and (c) antigens that are ectopically expressed. Somatic mutations can increase immunogenicity by producing new antigenic epitopes via point mutations, frame shifts, or coding sequence extensions or truncations [95]. Several techniques are used for their detection, including serological analysis of tumor antigens by recombinant cDNA expression cloning (SEREX), phage display, serological proteome analysis (SERPA), multiple affinity protein profiling (MAPPing), and protein microarrays [97]. 

Currently, there are some candidate autoantibodies as clinically useful biomarkers for gastric cancer; namely, anti-p53, anti-carcinoembryonic antigen (CEA), anti-mucin, anti-survivin, and anti-livin autoantibodies.

p53 is a tumor suppressor gene that plays a critical role in oncology. Its protein participates in the regulation of the cell-cycle, acts as a transcriptional transactivator/repressor, helps in DNA repair, suppresses cell growth, induces apoptosis and has many other functions [98]. The production of anti-p53 autoantibodies is strongly related to p53 protein overexpression in the tumor tissue [99]. Autoantibodies against the p53 protein were detected for the first time in sera of patients with breast cancer [100] and then in many other solid tumors. In gastric cancer, 20% of all patients and 46% of patients with p53-positive tumors have high levels of anti-p53 antibodies [101]. Regardless of the moderate sensitivity, there is consensus on the very high specificity (around 96%) of p53 antibodies for malignancy [102,103]. Several studies have also demonstrated that anti-p53 antibodies are more prevalent in advanced gastric cancers with a prevalence of regional lymph node involvement [95,101,104,105] recognizing the poor prognostic value of p53 autoantibody markers in gastric carcinoma.

Antibodies to CEA, an oncofetal glycoprotein commonly measured as a tumor marker, may be found in 46–56% of gastrointestinal tumors, especially in cancer at an early stage, even with undetectable circulating CEA [7,106]. However, they are also found in 10% of healthy individuals suggesting they could be part of the natural autoantibody repertoire. Anti-CEA antibodies are associated with the host immune response against the tumor and show a good prognostic value for survival [99,107]. Antibodies to mucin [108], surviving, and livin [109] have also been detected in patients with gastric cancer, with a prevalence of 75%, 40%, and 50%, respectively. They could represent new tumor markers not only for diagnosis but also for postoperative monitoring of gastric cancer patients, particularly in those lacking anti-p53 antibodies [95]. 

Autoantibodies to the extracellular protein kinase A (ECPKA), a cAMP-dependent intracellular enzyme, are markedly up-regulated in the sera of cancer patients, have been found in many malignant tumors, including gastric cancer. Although these antibodies measure malignant transformation in all cells and are not specific to one type of cancer, they have a sensitivity of 90% with a specificity of 87% and could be used as a universal screening method to detect serum tumor markers [110].

However, notwithstanding their high diagnostic specificity, in clinical practice, autoantibody response has been seen to be highly variable from patient to patient, probably due to diverse immune responses resulting from the highly heterogeneous nature of cancer and inherent genotypic (and epigenetic) variations within a population [95]. In addition, contrary to what occurs in autoimmune diseases, assays that measure a single tumor-associated autoantibody appear to have little diagnostic use for cancer due to their low frequency, rarely exceeding 30%. A possible strategy for overcoming this limitation due to individual variability and poor diagnostic sensitivity could be combining known autoantibody markers with other biomarkers for gastric cancer, such as tumor markers like carcinoembryonic antigen (CEA) [111], CA19-9 [112], and CA72-4 [113] markers related to chronic atrophic gastritis (e.g., parietal cell antibodies, *H. pylori* antibodies and serum pepsinogens I and II, gastrin [114]), microRNAs [115] or glycosylation signatures [116].

Another strategy to increase diagnostic sensitivity is to associate multiple antibody markers. To this end, Werner et al. studied 329 gastric cancer patients, 321 healthy controls and 124 participants with other diseases of the upper digestive tract by multiplex serology using a fluorescent bead-based glutathione S-transferase (GST) capture immunosorbent assay [117]. Among 64 candidate autoantibodies directed against gastric tumor-associated antigens, they identified five antibodies: MAGEA4, CTAG1, TP53, ERBB2_C, and SDCCAG8. At 98% specificity, sensitivity for gastric cancer detection for single antibodies was not higher than 12%, while a combination of the five antibodies enabled recognition of 32% of early-stage gastric cancer with a specificity of 87% [117]. 

Using an ELISA assay to detect autoantibodies towards an antigenic panel containing a seven-marker combination (p53, Koc, p62, c-myc, IMP1, survivin and p16), in a cohort of 383 patients (88 with gastric adenocarcinoma, 79 with gastric dysplasia, 76 with chronic atrophic gastritis, and 140 individuals with normal gastric mucosa), Zhou et al. reported a sensitivity of 64% for adenocarcinoma with a specificity of 87%. The area under the receiver operating characteristic (ROC) curve was 0.730. Sensitivity for gastric cancer did not increase with the addition of other autoantibodies to tumor-associated antigens [118].

In a similar study by Wang and coworkers, autoantibodies against eight tumor-associated recombinant antigens (IMP1, p62, Koc, p53, c-myc, cyclin B1, survivin and p16) determined by ELISA and Western blot, showed 56.1% sensitivity for gastric cancer detection, at 86.2% specificity. The highest frequency (27%) was found for cyclin B1 [119].

Thus, a substantial number of autoantibodies present in patients with gastric cancer have been identified. Although some of the autoantibodies are highly specific, their low diagnostic sensitivity has limited their application in clinical practice and assays that measure a single tumor-associated autoantibody appear to have little diagnostic utility for cancer detection. In the future, availability of new multiplex technology for the simultaneous detection of many autoantibodies might prove to be able to overcome these limitations by providing cancer-specific autoantibody profiles to be used for population screening for the early detection of gastric cancer.

## 4. Conclusions

There is evidence that the incidence of gastric neoplasms is higher in patients with autoimmune gastritis compared to the general population. Many studies in humans and in mouse models of gastritis indicate that chronic inflammation stimulates gastric cells to produce inflammatory cytokines which play a relevant role in regulating oxyntic atrophy, hyperplasia, metaplasia, and progression to gastric cancer by up-regulating expression of progenitor cells. Recent data on gastric cancer stem cell involvement may provide insights into the molecular pathway of carcinogenesis, eventually leading to development of new therapeutic approaches to target early-stage gastric cancer.

## Figures and Tables

**Table 1:** Clinical presentation, serology, pathology and neoplastic risk of autoimmune gastritis.

**Clinical Presentation**	No symptoms or dyspepsia	
	Anemia (iron deficiency, vitamin B12 deficiency)	
	Coexisting autoimmune diseases:	Autoimmune thyroid diseases (Hashimoto and Graves)
		Type 1 diabetes
		Addison disease
		Polyglandular autoimmune syndromes type III
Serology	Gastrin 17	>10 pmol/L
	Pepsinogen I	<30 μg/L
	Pepsinogen II	normal (3–15 μg/L)
	Parietal cell autoantibodies	pos 90–95%
	Intrinsic factor autoantibodies	pos 30–50%
Pathology	Corpus/fundus restricted gastritis	
Neoplastic Risk	Gastric carcinoid: increased according to gastric (oxyntic) atrophy score to the corpus and fundus of the stomach	
	Gastric adenocarcinoma: increased according to pangastric atrophy score	

## References

[1] Burnham T.K. (1972). Antinuclear antibodies in patients with malignancies. Lancet.

[2] Zermosky J.O., Gormy M.K., Jarczewska K. (1972). Malignancy associated with antinuclear antibodies. Lancet.

[3] Solans-Laqué R., Pérez-Bocanegra C., Salud-Salvia A., Fonollosa-Plá V., Rodrigo M.J., Armadans L., Simeón-Aznar C.P., Vilardell-Tarres M. (2004). Clinical significance of antinuclear antibodies in malignant diseases: Association with rheumatic and connective tissue paraneoplastic syndromes. Lupus.

[4] Atalay C., Atalay G., Yilmaz K.B., Altinok M. (2005). The role of anti-CENP-B and anti-SS-B antibodies in breast cancer. Neoplasma.

[5] Toubi E., Shoenfeld Y. (2007). protective autoimmunity in cancer. Oncol. Rep..

[6] Lv S., Zhang J., Wu J., Zheng X., Chu Y., Xiong S. (2005). Origin and anti-tumor effects of anti-dsDNA autoantibodies in cancer patients and tumor-bearing mice. Immunol. Lett..

[7] Konstadoulakis M.M., Syrigos K.N., Albanopoulos C., Mayers G., Golematis B. (1994). The presence of anti-carcinoembryonic antigen (CEA) antibodies in the sera of patients with gastrointestinal malignancies. J. Clin. Immunol..

[8] Schattner A., Shani A., Talpaz M., Bentwich Z. (1983). Rheumatoid factors in the sera of patients with gastrointestinal carcinoma. Cancer.

[9] Betterle C., Peserico A., Bersani G., Ninfo V., del Prete G.F., Stefani R., Nitti D. (1979). Circulating antibodies in malignant melanoma patients. Dermatologica.

[10] Molander S., Jønsson V., Andersen L.P., Bennetzen M., Christiansen M., Hou-Jensen K., Madsen H.O., Ryder L.P., Permin H., Wiik A. (2000). Pseudolymphoma and ventricular maltoma in patients with chronic gastritis, ulcer and Helicobacter pylori infection. Ugeskr. Laeger.

[11] Tomer Y., Shoenfeld Y., Shoenfeld Y., Gershwin M.E. (2000). Autoantibodies, autoimmunity and cancer. Cancer and Autoimmunity.

[12] Bradford-Hill A. (1965). The environment and disease: Association or causation?. Proc. R. Soc. Med..

[13] Mellemkjaer L., Andersen V., Linet M.S., Gridley G., Hoover R., Olsen J.H. (1997). Non-Hodgkin’s lymphoma and other cancers among a cohort of patients with systemic lupus erythematosus. Arthritis Rheum..

[14] Valesini G., Priori R., Bavoillot D., Osborn J., Danieli M.G., del Papa N., Gerli R., Pietrogrande M., Sabbadini M.G., Silvestris F. (1997). Differential risk of non-Hodgkin’s lymphoma in Italian patients with primary Sjӧgren’s syndrome. J. Rheumatol..

[15] Villa A.R., Kraus A., Alarcon-Segovia D., Shoenfeld Y., Gershwin M.E. (2000). Autoimmune rheumatic diseases and cancer: Evidence of causality?. Cancer and Autoimmunity.

[16] Moinzadeh P., Fonseca C., Hellmich M., Shah A.A., Chighizola C., Denton C., Ong V.H. (2014). Association of anti-RNA polymerase III autoantibodies and cancer in scleroderma. Arthritis Res. Ther..

[17] Toh BH. (2014). Diagnosis and classification of autoimmune gastritis. Autoimmun. Rev..

[18] Marignani M., Delle Fave G., Mecarocci S., Bordi C., Angeletti S., D’Ambra G., Aprile M.R., Corleto V.D., Monarca B., Annibale B. (1999). High prevalence of atrophic body gastritis in patients with unexplained microcytic and macrocytic anemia. Am. J. Gastroenterol..

[19] Bizzaro N., Antico A. (2014). Diagnosis and classification of pernicious anemia. Autoimmun. Rev..

[20] Weis V.G., Goldenring J.R. (2009). Current understanding of SPEM and its standing in the preneoplastic process. Gastric Cancer.

[21] Kokkola A., Sjoblom S.M., Haapiainen R., Sipponen P., Puolakkainen P., Jarvinen H. (1998). The risk of gastric carcinoma and carcinoid tumours in patients with pernicious anemia: A prospective follow-up study. Scand. J. Gastroenterol..

[22] Dixon M.F., Genta R.M., Yardley J.H., Correa P. (1996). Classification and grading of gastritis. The updated Sydney System. International Workshop on the Histopathology of Gastritis, Houston 1994. Am. J. Surg. Pathol..

[23] Hawa M., Beyan H., Leslie R.D. (2004). Principles of autoantibodies as disease-specific markers. Autoimmunity.

[24] Weck M.N., Brenner H. (2006). Prevalence of chronic atrophic gastritis in different parts of the world. Cancer Epidemiol. Biomarkers Prev..

[25] Weetman A.P. (2005). Non-thyroid antibodies in autoimmune thyroid disease. Best Pract. Res. Clin. Endocrinol. Metab..

[26] Van den Driessche A., Eenkhoorn V., van Gaal L., de Block C. (2009). Type 1 diabetes and autoimmune polyglandular syndrome: A clinical review. Neth. J. Med..

[27] Betterle C., Presotto F., Walker S.A., Jara L.J. (2008). Autoimmune polyendocrine syndromes (APS) or multiple autoimmune syndromes (MAS). Handbook of Systemic Autoimmune Diseases. Endocrine Manifestations of Systemic Autoimmune Diseases.

[28] Strickland R.G., Mackay I.R. (1973). The reappraisal of the nature and significance of chronic atrophic gastritis. Am. J. Dig. Dis..

[29] Toh B.H., Sentry J.W., Alderuccio F. (2000). The causative H^+^/K^+^ ATPase antigen in the pathogenesis of autoimmune gastritis. Immunol. Today.

[30] Weck M.N., Brenner H. (2008). Association of Helicobacter pylori infection with chronic atrophic gastritis: Meta-analyses according to type of disease definition. Int. J. Cancer.

[31] Ma J.Y., Borch K., Sjostrand S.E., Janzon L., Mardh S. (1994). Positive correlation between H, K-adenosine triphosphatase autoantibodies and Helicobacter pylori antibodies in patients with pernicious anemia. Scand. J. Gastroenterol..

[32] Faller G., Kirchner T. (2005). Immunological and morphogenic basis of gastric mucosa atrophy and metaplasia. Virchows. Arch..

[33] Claeys D., Faller G., Appelmelk B., Negrini R., Kirchner T. (1998). The gastric H^+^/K^+^ ATPase is a major autoantigen in chronic Helicobacter pylori gastritis with body mucosa atrophy. Gastroenterology.

[34] Amedei A., Bergman M.P., Appelmelk B., Azzurri A., Benagiano M., Tamburini C., van der Zee R., Telford J.L., Vandenbroucke-Grauls C.M.J.E., D’Elios M.M. (2003). Molecular mimicry between Helicobacter pylori antigens and H^+^K^+^-adenotriphosphatase in human gastric autoimmunity. J. Exp. Med..

[35] D’Elios M.M., Appelmelk B.J., Amedei A., Bergman M.P., Del Prete G.F. (2004). Gastric autoimmunity: The role of Helicobacter pylori and molecular mimicry. Trends Mol. Med..

[36] Plebani M., Basso D., Tozzoli R., Bizzaro N., Villalta D., Tonutti E., Pinchera A. (2009). Le malattie autoimmuni del tratto gastro-enterico. Il Laboratorio Nelle Malattie Autoimmuni D’organo.

[37] Faller G., Winter M., Steininger H., Lehn N., Meining A., Bayerdorffer E., Kirchner T. (1999). Decrease of antigastric autoantibodies in Helicobacter pylori gastritis after cure of infection. Pathol. Res. Pract..

[38] Ohkusa T., Fujiki K., Takashimizu I., Kuma G.A.J., Tanizawa T., Eishi Y., Yokoyama T., Watanabe M. (2001). Improvement in atrophic gastritis and intestinal metaplasia in patients in whom Helicobacter pylori was eradicated. Ann. Intern. Med..

[39] Oksanen A., Sipponen P., Karttunen R., Miettinen A., Veijola L., Sarna S., Rautelin H. (2000). Atrophic gastritis and Helicobacter pylori infection in outpatients referred for gastroscopy. Gut.

[40] De Block C.E., de Leeuw I.H., Bogers J.J., Pelckmans P.A., Ieven M., van Marck E.A., van Hoof V., Máday E., van Acker K.L., van Gaal L.F. (2002). Helicobacter pylori, parietal cell antibodies and autoimmune gastropathy in type 1 diabetes mellitus. Aliment. Pharmacol. Ther..

[41] Annibale B., Aprile M.R., D’Ambra G., Caruana P., Bordi C., Delle Fave G. (2000). Cure of Helicobacter pylori infection in atrophic body gastritis patients does not improve mucosal atrophy but reduces hypergastrinemia and its related effects on body ECL-cell hyperplasia. Aliment. Pharmacol. Ther..

[42] D’Elios M.M., Bergman M.P., Azzurri A., Amedei A., Benagiano M., de Pont J.J., Cianchi F., Vandenbroucke-Grauls C.M., Romagnani S., Appelmelk B.J. (2001). H(^+^),K(^+^)- ATPase (proton pump) is the target autoantigen of Th1-type cytotoxic T cells in autoimmune gastritis. Gastroenterology.

[43] Vergelli M., Hemmer B., Muraro P.A., Tranquill L., Biddison W.E., Sarin A., McFarland H.F., Martin R. (1997). Human autoreactive CD4 T cell clones use perforin or Fas/Fas ligand-mediated pathways for target cell lysis. J. Immunol..

[44] De Block C.E.M., de Leeuw I.H., van Gaal L.F. (2008). Autoimmune gastritis in type 1 diabetes: A clinically oriented review. J. Clin. Endocrinol. Metab..

[45] Alderuccio F., Sentry J.W., Marshall A.C., Biondo M., Toh B.H. (2002). Animal models of human disease: Experimental autoimmune gastritis and pernicious anemia. Clin. Immunol..

[46] Toh B.H., Van Driel I.R., Gleeson P.A. (1997). Mechanisms of disease: Pernicious anemia. N. Engl. J. Med..

[47] Callaghan J.M., Khan M.A., Alderuccio F., van Driel I.R., Gleeson P.A., Toh B.H. (1993). Alpha and beta subunits of the gastric H^+^/K^+^-ATPase are concordantly targeted by parietal cell autoantibodies associated with autoimmune gastritis. Autoimmunity.

[48] Asano M., Toda M., Sakaguchi N., Sakaguchi S. (1996). Autoimmune disease as a consequence of developmental abnormality of a T cell subpopulation. J. Exp. Med..

[49] Taguchi O., Takahashi T. (1996). Administration of anti-interleukin-2 receptor alfa antibody in vivo induces localized autoimmune disease. Eur. J. Immunol..

[50] Zittoun J. (2001). Biermer’s disease. Rev. Prat..

[51] Toh B.H., Alderuccio F. (2004). Pernicious anaemia. Autoimmunity.

[52] Toh B.H., Chan J., Kyaw T., Alderuccio F. (2012). Cutting edge issues in autoimmune gastritis. Clin. Rev. Allergy Immunol..

[53] Antico A., Tozzoli R., Bizzaro N., Villalta D., Tonutti E., Pinchera A. (2009). L’autoimmunità gastrica. Il Laboratorio Nelle Malattie Autoimmuni D’organo.

[54] Antico A., Tampoia M., Villalta D., Tonutti E., Tozzoli R., Bizzaro N. (2012). Clinical usefulness of the serological gastric biopsy for the diagnosis of chronic autoimmune gastritis. Clin. Dev. Immunol..

[55] Tozzoli R., Kodermaz G., Perosa A.R., Tampoia M., Zucano A., Antico A., Bizzaro N. (2010). Autoantibodies to parietal cells as predictors of atrophic body gastritis: A five-year prospective study in patients with autoimmune thyroid diseases. Autoimmun. Rev..

[56] Seetharam B., Alpers D.H., Allen R.H. (1981). Isolation and characterization of the ileal receptor for intrinsic factor-cobalamin. J. Biol. Chem..

[57] Carmel R. (1992). Reassessment of the relative prevalence of antibodies to gastric parietal cell and to intrinsic factor in patients with pernicious anaemia: Influence of patient age and race. Clin. Exp. Immunol..

[58] Conn D.A. (1986). Detection of type I and II antibodies to intrinsic factor. Med. Lab. Sci..

[59] Vannella L., Sbrozzi-Vanni A., Lahner E., Bordi C., Pilozzi E., Corleto V.D., Osborn J.F., Delle F.G., Annibale B. (2011). Development of type I gastric carcinoid in patients with chronic atrophic gastritis. Aliment. Pharmacol. Ther..

[60] Vannella L., Lahner E., Osborn J., Annibale B. (2013). Systematic review: Gastric cancer incidence in pernicious anaemia. Aliment Pharmacol. Ther..

[61] Landgren A.M., Landgren O., Gridley G., Dores G.M., Linet M.S., Morton L.M. (2011). Autoimmune disease and subsequent risk of developing alimentary tract cancers among 4.5 million US male veterans. Cancer.

[62] Hemminki K., Liu X., Ji J., Sundquist J., Sundquist K. (2012). Effect of autoimmune diseases on mortality and survival in subsequent digestive tract cancers. Ann. Oncol..

[63] Nguyen T.L., Khurana S.S., Bellone C.J., Capoccia B.J., Sagartz J.E., Kesman R.A., Mills J.C., DiPaolo R.J. (2013). Autoimmune gastritis mediated by CD4^+^ T cells promotes the development of gastric cancer. Cancer Res..

[64] Dinis-Ribeiro M., Areia M., de Vries A.C., Marcos-Pinto R., Monteiro-Soare M., O’Connor A., Pereira C., Pimentel-Nunes P., Correia R., Ensari A. (2012). Management of precancerous conditions and lesions in the stomach (MAPS): Guideline from European Society of Gastrointestinal Endoscopy (ESGE), European Helicobacter Study Group (EHSG), European Society of Pathology (ESP), and the Sociedade Portoguesa de Endoscopia Digestiva (SPED). Virchows. Arch..

[65] Rugge M., Correa P., di Mario F., El-Omar E., Fiocca R., Geboes K., Genta R.M., Graham D.Y., Hattori T., Malfertheiner P. (2008). OLGA staging for gastritis: A tutorial. Dig. Liver. Dis..

[66] El Zimaity H.M., Ota H., Graham D.Y., Akamatsu T., Katsuyama T. (2002). Patterns of gastric atrophy in intestinal type gastric carcinoma. Cancer.

[67] Epplein M., Xiang Y.B., Cai Q., Peek R.M., Lin H., Correa P., Gao J., Wu J., Michel A., Pawlita M. (2013). Circulating cytokines and gastric cancer risk. Cancer Causes Control..

[68] Lida T., Iwahashi M., Katsuda M., Nakamori M., Nakamura M., Naka T., Ojima T., Ueda K., Hayata K., Nakamura Y. (2011). Tumor-infiltrating CD4^+^ Th 17 cells produce IL-17 in tumor microenvironment and promote tumor progression in human gastric cancer. Oncol. Rep..

[69] Meng X.Y., Zhou C.H., Ma J., Jiang C., Ji P. (2012). Expression of interleukin-17 and its clinical significance in gastric cancer patients. Med. Oncol..

[70] Dai Z.M., Zhang T.S., Lin S., Zhang W.G., Liu D., Cao X.M., Li H.B., Wang M., Liu X.H., Liu K. (2016). Role of IL-17A rs2275913 and IL-17F rs763780 polymorphisms in risk of cancer development: An update meta-analysis. Sci. Rep..

[71] Muruyama T., Kono K., Mizukami Y., Kawaguchi Y., Mimura K., Watanabe M., Izawa S., Fujii H. (2010). Distribution of Th17 cells and FoxP3(+) regulatory T cells in tumor-infiltrating lymphocytes, tumor-draining lymph nodes and peripheral blood lymphocytes in patients with gastric cancer. Cancer Sci..

[72] Kuai W.X., Wang Q., Yang X.Z., Zhao Y., Yu R., Tang X.J. (2012). Interleukin-8 associates with adhesion, migration, invasion and chemosensitivity of human gastric cancer cells. World J. Gastroenterol..

[73] Bockerstett K.A., DiPaolo R.J. (2017). Regulation of gastric carcinogenesis by inflammatory cytokines. Cell Mol. Gastroenterol. Hepatol..

[74] Huang F.Y., Chan A.O., Rashid A., Wong D.K., Seto W.K., Cho C.H., Lai C.L., Yuen M.F. (2016). Interleukin 1β increases the risk of gastric cancer through induction of aberrant DNA methylation in a mouse model. Oncol. Lett..

[75] Al-Sammak F., Kalinski T., Winert S., Link A., Wex T., Malfertheiner P. (2013). Gastric epithelial expression of IL-12 cytokine family in Helicobacter pylori infection in human: Is it head or tail of the coin?. PLoS ONE.

[76] Tsai C.Y., Wang C.S., Tsai M.M., Chi H.C., Cheng W.L., Tseng Y.H., Chen C.Y., Lin C.D., Wu J.I., Wang L.H. (2014). Interleukin-32 increase human gastric cancer cell invasion associated with tumor progression and metastasis. Clin. Cancer Res..

[77] Buzzelli J.N., Chalinor H.V., Pavlic D.I., Sutton P., Menheniott T.R., Giraud A.S., Judd L.M. (2015). IL33 is a stomach alarmin that initiates a skewed Th2 response to injury and infection. Cell. Mol. Gastroenterol. Hepatol..

[78] Lahner E., Esposito G., Galli G., Annibale B. (2015). Atrophic gastritis and pre-malignant gastric lesions. Transl. Gastrointest. Cancer.

[79] Schneller J., Gupta R., Mustafa J., Villanueva R., Straus E.W., Raffaniello R.D. (2006). Helicobacter pylori infection is associated with a high incidence of intestinal metaplasia in the gastric mucosa of patients at inner-city hospitals in New York. Dig. Dis. Sci..

[80] Wong B.C., Lam S.K., Wong W.M., Chen J.S., Zheng T.T., Feng R.E., Lai K.C., Cheng W.H., Yuen S.T., Leung S.Y. (2004). Helicobacter pylori eradication to prevent gastric cancer in a high-risk region of China: A randomized controlled trial. JAMA.

[81] Yamaoka Y. (2010). Mechanisms of disease: Helicobacter pylori virulence factors. Nat. Rev. Gastroenterol. Hepatol..

[82] Yong X., Tang B., Li B.-S., Xie R., Hu C.J., Luo G., Qin Y., Dong H., Yang S.M. (2015). Helicobacter pylori virulence factor CagA promotes tumorigenesis of gastric cancer via multiple signaling pathways. Cell Commun. Signal..

[83] Van Doorn L.J., Figueiredo C., Sanna R., Plaisier A., Schneeberger P., de Boer W., Quint W. (1998). Clinical relevance of the cagA, vacA, and iceA status of Helicobacter pylori. Gastroenterology.

[84] Correa P., Piazuelo M.B. (2012). The gastric precancerous cascade. J. Dig. Dis..

[85] Elsborg L., Mosbech J. (1979). Pernicious anaemia as a risk factor in gastric cancer. Acta Med. Scand..

[86] Nikou G.C., Angelopoulos T.P. (2012). Current concepts on gastric carcinoid tumors. Gastroenterol. Res. Pract..

[87] Vanoli A., La Rosa S., Luinetti O., Klersy C., Manca R., Alvisi C., Rossi S., Trespi E., Zangrandi A., Sessa F. (2013). Histologic changes in type A chronic atrophic gastritis indicating increased risk of neuroendocrine tumor development: The predictive role of dysplastic and severely hyperplastic enterochromaffin-like cell lesions. Hum. Pathol..

[88] Zhou K., Ho W., Pisegna R.J. (2015). Gastric carcinoids: Classification and Diagnosis. Management of Pancreatic Neuroendocrine Tumors.

[89] Burkitt M.D., Pritchard D.M. (2006). Review article: Pathogenesis and management of gastric carcinoid tumours. Aliment Pharmacol. Ther..

[90] Minalyan A., Benhammou N.J., Artashesyan A., Lewis S.M., Pisegna J.R. (2017). Autoimmune atrophic gastritis: Current perspectives. Clin. Exp. Gastroenterol..

[91] Visvader J.E. (2011). Cells of origin in cancer. Nature.

[92] Shibata W., Sue S., Tsumura S., Ishii Y., Sato T., Kameta E., Sugimori M., Yamada H., Kaneko H., Sasaki T. (2017). Helicobacter-induced gastric inflammation alters the properties of gastric tissue stem/progenitor cells. BMC Gastroenterol..

[93] Tan E.M. (2001). Autoantibodies as reporters identifying aberrant cellular mechanisms in tumorigenesis. J. Clin. Investig..

[94] Werner S., Chen H., Tao S., Brenner H. (2015). Systematic review: Serum autoantibodies in the early detection of gastric cancer. Int. J. Cancer.

[95] Macdonald I.K., Parsy-Kowalska C.B., Chapman C.J. (2017). Autoantibodies: Opportunities for early cancer detection. Trends Cancer.

[96] Liu W., Peng B., Lu Y., Xu W., Qian W., Zhang J.Y. (2011). Autoantibodies to tumor-associated antigens as biomarkers in cancer immunodiagnosis. Autoimmun. Rev..

[97] Zaenker P., Ziman M.R. (2013). Serologic autoantibodies as diagnostic cancer biomarkers—A review. Cancer Epidemiol. Biomarkers Prev..

[98] Flammann H.T., Kuhn HM., Shoenfeld Y., Gershwin M.E. (2000). P53 autoantibodies and cancer: Specificity, diagnosis and monitoring. Cancer and Autoimmunity.

[99] Saif M.W., Zalonis A., Syrigos K. (2007). The clinical significance of autoantibodies in gastrointestinal malignancies: An overview. Expert Opin. Biol. Ther..

[100] Crawford L.V., Pim D.C., Bulbrook R.D. (1982). Detection of antibodies against the cellular protein p53 in sera from patients with breast cancer. Int. J. Cancer.

[101] Wurl P., Weigmann F., Meye A., Fittkau M., Rose U., Berger D., Rath F.W., Dralle H., Taubert H. (1997). Detection of p53 autoantibodies in sera of gastric cancer patients and their prognostic relevance. Scand. J. Gastroenterol..

[102] Soussi T. (2000). p53 Antibodies in the sera of patients with various types of cancer: A review. Cancer Res..

[103] Shimada H., Ochiai T., Nomura F. (2003). Japan p53 Antibody Research Group. Titration of serum p53 antibodies in 1085 patients with various types of malignant tumors: A multiinstitutional analysis by the Japan p53 Antibody Research Group. Cancer.

[104] Shiota G., Ishida M., Noguchi N., Takano Y., Oyama K., Okubo M., Katayama S., Harada K., Hori K., Ashida K. (1998). Clinical significance of serum P53 antibody in patients with gastric cancer. Res. Commun. Mol. Pathol. Pharmacol..

[105] Maehara Y., Kakeji Y., Watanabe A., Baba H., Kusumoto H., Kohnoe S., Sugimachi K. (1999). Clinical implications of serum anti-p53 antibodies for patients with gastric carcinoma. Cancer.

[106] Ura Y., Ochi Y., Hamazu M., Ishida M., Nakajima K., Watanabe T. (1985). Studies on circulating antibody against carcinoembryonic antigen (CEA) and CEA-like antigen in cancer patients. Cancer Lett..

[107] Albanopoulos K., Armakolas A., Konstadoulakis M.M., Leandros E., Tsiompanou E., Katsaragakis S., Alexiou D., Androulakis G. (2000). Prognostic significance of circulating antibodies against carcinoembryonic antigen (anti-CEA) in patients with colon cancer. Am. J. Gastroenterol..

[108] Nakamura H., Hinoda Y., Nakagawa N., Makiguchi Y., Itoh F., Endo T., Imai K. (1998). Detection of circulating anti-MUC1 mucin core protein antibodies in patients with colorectal cancer. J. Gastroenterol..

[109] Yagihashi A., Asanuma K., Nakamura M., Araya J., Mano Y., Torigoe T., Kobayashi D., Watanabe N. (2001). Detection of anti-survivin antibody in gastrointestinal cancer patients. Clin. Chem..

[110] Cho-Chung Y.S. (2006). Autoantibody biomarkers in the detection of cancer. Biochim. Biophys. Acta..

[111] Qiu L.L., Hua P.Y., Ye L.L., Wang Y.C., Qiu T., Bao H.Z., Wang L. (2007). The detection of serum anti-p53 antibodies from patients with gastric carcinoma in China. Cancer Detect Prev..

[112] Shimizu K., Ueda Y., Yamagishi H. (2005). Titration of serum p53 antibodies in patients with gastric cancer: A single-institute study of 40 patients. Gastric Cancer.

[113] Shimada H., Noie T., Ohashi M., Oba K., Takahashi Y. (2014). Clinical significance of serum tumor markers for gastric cancer: A systematic review of literature by the Task Force of the Japanese Gastric Cancer Association. Gastric Cancer.

[114] Di Mario F., Cavallaro L.G. (2008). Non-invasive tests in gastric diseases. Dig. Liver Dis..

[115] Majeed W., Iftikhar A., Khaliq T., Aslam B., Muzaffar H., Atta K., Mahmood A., Waris S. (2016). Gastric carcinoma: Recent trends in diagnostic biomarkers and molecular targeted therapies. Asian Pac. J. Cancer Prev..

[116] Zayakin P., Ancāns G., Siliņa K., Meistere I., Kalniņa Z., Andrejeva D., Endzeliņš E., Ivanova L., Pismennaja A., Ruskule A. (2013). Tumor-associated autoantibody signature for the early detection of gastric cancer. Int. J. Cancer.

[117] Werner S., Chen H., Butt J., Michel A., Knebel P., Holleczek B., Zörnig I., Eichmüller S.B., Jäger D., Pawlita M. (2016). Evaluation of the diagnostic value of 64 simultaneously measured autoantibodies for early detection of gastric cancer. Sci. Rep..

[118] Zhou S.L., Ku J.W., Fan Z.M., Yue W.B., Du F., Zhou Y.F. (2015). Detection of autoantibodies to a panel of tumor-associated antigens for the diagnosis values of gastric cardia adenocarcinoma. Dis. Esophagus.

[119] Wang P., Song C., Xie W., Ye H., Wang K., Dai L., Zhang Y., Zhang J. (2014). Evaluation of diagnostic value in using a panel of multiple tumor-associated antigens for immunodiagnosis of cancer. J. Immunol. Res..

